# Imaging-derived biological age across multiple organs links to mortality and aging-related health outcomes

**DOI:** 10.1038/s41514-026-00377-7

**Published:** 2026-04-03

**Authors:** Veronika Ecker, Bin Yang, Sergios Gatidis, Thomas Küstner

**Affiliations:** 1 Institute of Signal Processing and System Theory, University of Stuttgart, Stuttgart, Germany; 2 Diagnostic and Interventional Radiology, University Hospital Tübingen, Tübingen, Germany

**Keywords:** Biomarkers, Computational biology and bioinformatics, Health care, Medical research

## Abstract

Aging is a complex, multifactorial process, influencing disease risk and overall health. While chronological age (CA) is widely used in clinical practice, it fails to capture individual aging trajectories. Current approaches to estimate biological age (BA) often focus on single organs or predefined clinical biomarkers, limiting comprehensive assessment. We introduce a novel, purely imaging-driven deep learning framework for organ-specific BA estimation across seven organ systems. Our uncertainty-aware ResNet-based models autonomously learned aging-related features from imaging data in 70,000 UK Biobank participants, eliminating manual feature selection biases. Training on a healthy cohort, where CA approximates BA, allows learning normative aging patterns. When applied to a broader cohort, deviations from typical aging indicate older or younger BA. Our findings demonstrate the feasibility of BA estimation, even in organs with subtle aging features. While aging is largely heterogeneous across organs, we also identified correlations in aging patterns. We further showed that accelerated aging is prognostic of mortality and health outcomes, offering insights for personalized assessments.

## Introduction

Human aging is a complex process influenced by biological, environmental, and socio-economic factors, including lifestyle, access to healthcare, and overall health status. These factors drive gradual structural and functional changes in cells, tissues, and organs^1^. Such alterations are closely linked to the development of many prevalent diseases, including cardiovascular disorders^2,3^, neurodegenerative conditions like Alzheimer’s disease^4,5^, or metabolic diseases such as type 2 diabetes^6,7^. In clinical practice, characteristic age distributions of diseases help to estimate their likelihood in patients, significantly influencing diagnostic decisions and treatment strategies. Moreover, the growing longevity trend, reflected in increasing life expectancy across many populations, amplifies the importance of understanding age-related diseases and adapting healthcare systems accordingly^8^.

Because these structural and functional changes accumulate over time, chronological age (CA) is commonly used in medicine as an indicator of aging. However, while CA provides a standardized measure, it does not fully capture the biological processes underlying aging, nor does it account for individual variability in age-related phenotypes among individuals of the same chronological age group. The concept of biological age (BA) was introduced as a biomarker to better reflect the true individual variation associated with aging. However, the quantification of BA remains challenging as there is no straightforward definition or ground truth, given the multitude and complexity of influencing parameters.

To address these challenges, conventional methods have primarily relied on clinical biomarkers such as DNA methylation^9,10^, transcriptomics^11,12^, functional assessments^13,14^, laboratory data^15,16^, and patient histories^17^ to extract aging-related information. Extensive research focuses on improving these approaches, aiming to develop biomarkers that not only correlate strongly with CA but also demonstrate high prognostic power for age-related diseases and mortality. Yet, many of these methods rely on a single BA estimate for an individual, which may not fully reflect the heterogeneous nature of aging across different organs and tissues. To arrive at a more comprehensive assessment, investigations into BA at the multi-organ level are essential. Recent research by *Tian* et al. explored the BA estimation across multiple organ systems based on purely physiological and blood-derived phenotypes as well as phenotypes derived from brain imaging^18^. Individual aging clocks for seven body systems and three brain systems were developed.

The limited amount of available composite clinical biomarkers is usually insufficient to capture all age-related processes. Instead of directly quantifying changes at the cellular or molecular level, imaging-based approaches focus on detecting the observable consequences of the aging process in medical images. However, the information complexity and density demand the need for a systematic and objective analysis. Deep learning is particularly strong in identifying patterns at large-scale, and in addition, eliminates the need for hand-crafted features. Deep learning approaches have attracted attention and shown promising results in predicting the age of individual organs like the brain^19–23^, cardiovascular system^24,25^, knee^26,27^, chest^28,29^, and abdomen^30^. Since biological age (BA) is not directly observable in imaging data, most approaches use supervised learning to establish a link to chronological age (CA). Training on a healthy reference cohort allows the model to learn normative aging patterns, which can then be used to identify deviations as accelerated or decelerated aging in broader populations. Given this indirect link, it is equally important to assess how reliable such predictions are. Approaches such as quantifying prediction uncertainty or generating attention maps^19,31^ can provide insights into model confidence and highlight image regions most influential for the estimate.

Establishing normative aging patterns requires large and diverse reference groups, which are best represented in population-scale cohorts. Resources such as the UK Biobank (UKB)^32^ or the German National Cohort (NAKO)^33^ capture health data from the general population, in contrast to clinical studies that often target specific disease phenotypes. Their scale not only provides a robust basis for modeling biological age but also enables analyses across multiple organs, supporting comprehensive assessments, identification of factors shaping aging trajectories, and deeper insights into healthy aging and inter-organ relationships.

Despite these advantages, current imaging-based BA estimation faces several limitations and open challenges. First, even when trained on large healthy cohorts, supervised models remain vulnerable to biases if the selected reference groups do not adequately represent the diversity of the population. Second, while most studies estimate BA for individual organs, the inter-relationships between organs have not yet been systematically explored on the image level. Moreover, these imaging-derived BA estimates are rarely correlated with the rich set of outcomes available in large-scale cohorts such as UKB and NAKO. Although often referred to as an ‘aging clock’ or biomarker^18,34^, the prognostic value of imaging-derived BA remains uncertain, as many potential confounding factors still require systematic validation. In our own previous work^35^, we demonstrated the feasibility of multi-organ biological age estimation based on imaging data, providing preliminary evidence that deep learning can capture organ-specific aging trajectories. While these results highlighted the potential of imaging-derived multi-organ BA, they did not yet establish direct links to health outcomes or systematically address confounding effects in the training cohort. Addressing these gaps is essential to establishing reliable and generalizable aging trajectories across diverse populations, ultimately linking organ-level imaging signatures of aging to meaningful health outcomes.

We propose a deep learning-based framework for imaging-derived regional biological age estimation across multiple organ systems, enabling a comprehensive assessment of BA beyond single-organ models. Using an uncertainty-aware ResNet model^19,36^, we quantify the observable effect of aging from magnetic resonance images (MRI) and optical coherence tomography (OCT). The difference between predicted aging and CA, referred to as predicted age gaps (PAG), serves as an indicator for accelerated or decelerated aging. We further evaluate the prognostic value of the PAGs by relating them to outcomes associated with aging. Finally, by integrating multi-organ BA estimates, we explore the complex dynamics among different organ systems and factors underlying atypical aging, moving toward more reliable and holistic aging trajectories (code and models available at https://github.com/lab-midas/biological_age).

## Results

### Data characteristics and study overview

**Fig. 1:**
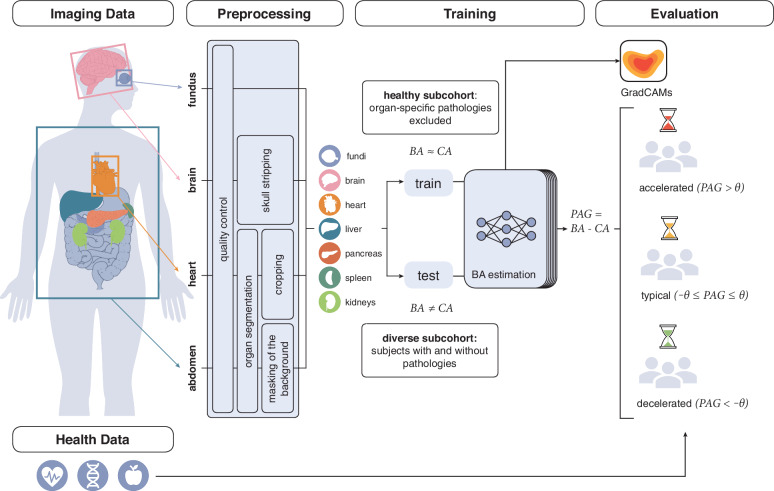
Organ-specific biological age (BA) estimation framework using magnetic resonance imaging (MRI) of the brain, heart, kidneys, liver, spleen, pancreas, and optical coherence tomography (OCT) scans of left and right fundus. Images of the brain, heart, abdomen, and fundus are first processed through an organ-specific preprocessing pipeline including quality control and organ segmentation. A ResNet-based age regression model is trained for each organ using healthy subjects with chronological age (CA) as reference. Predicted age gaps (PAGs), defined as the deviation between the predicted organ-specific BA and CA, are categorized into accelerated, typical, and decelerated aging based on the threshold *θ* derived from the cohort’s standard deviation for each organ. GradCAM attention maps highlight image regions contributing to BA predictions.

**Fig. 2:**
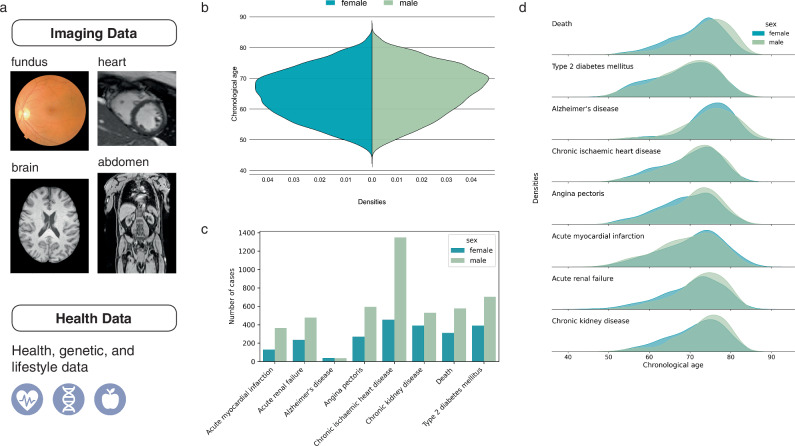
Overview of the dataset and cohort characteristics. Data composition (**a**) and cohort demographics are shown, including the age distribution of the full cohort (**b**) and the number of cases for relevant diseases (**c**) along with their respective age distributions (**d**). Only diagnoses recorded within the study period (from individual study entry to data retrieval in 01/2024) are included.

In this work, we predicted biological age (BA) using a deep learning model trained on imaging data for seven selected organs: brain, heart, kidneys (left and right), liver, pancreas, spleen, and fundus (left/right) (Fig. 1). Organs were chosen due to their susceptibility to age-related diseases and accessibility through advanced imaging techniques. We had access to 143,642 data samples (76,099 female/67,514 male) with CA ranging from 44 years to 83 years (mean CA = 64.23 years, median CA = 65 years) from the UKB. Imaging protocols included MRI sequences of the brain, cardiac, and abdomen, as well as OCT scans of the fundus. In addition to imaging data, the UKB contains extensive metadata, including health records, self-reported lifestyle factors, and genetic information. The cohort’s demographic distribution and disease prevalence provide context for the cohort composition and reveal notable sex-specific patterns, such as the over-representation of certain diseases in male subjects (Fig. 2).

Biological age predictions were derived from the available imaging data for each organ. As not all participants received every imaging scan, sample sizes vary by organ. Brain BA was estimated using 3D T1-weighted MRIs (*N* = 51,265, mean CA = 64.4 ± 7.78 years, 25,634 female), cardiac BA from cardiac cine MRIs (*N* = 68,359, mean CA = 65.41 ± 7.80 years, 36,702 female), and BA in abdominal organs using 3D Dixon scans (*N* = 70,193, mean CA = 65.4 ± 7.80 years, 36,119 female). Fundus BA was predicted using fundus OCT images (left: *N* = 87,310, mean CA = 57.65 ± 8.30 years, 45,305 female; right: *N* = 88,112, mean CA = 57.67 ± 8.30 years, 45,597 female). All images underwent manual quality control by expert readers. For deep learning analysis, imaging data were intensity normalized, and organ-specific segmentation models^37–39^ cropped images to the respective organs with background removal for input to the BA estimation model.

**Fig. 3:**
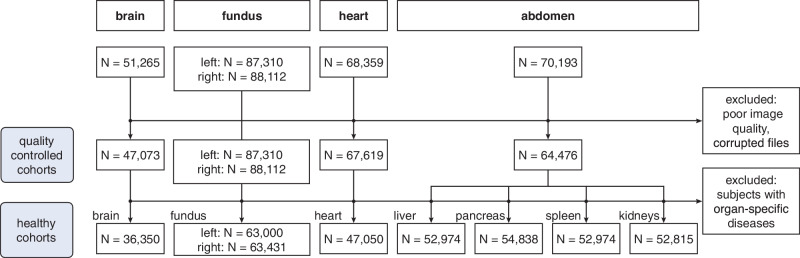
Cohort flowchart illustrating the exclusion process during preprocessing, including quality control and health-based selection, to define organ-specific healthy training cohorts for biological age estimation.

ResNet-based models were individually trained to capture each organ’s unique aging characteristics. Handcrafted features were not required, as the model learned organ-specific features related to aging directly from the imaging data, capturing complex structural (brain, abdomen, fundus) and functional (heart) changes. A healthy subcohort was used for training and validation, where *CA* ≈ *BA*, allowing CA to serve as a ground truth for supervised age regression. Only healthy participants without diagnosed diseases impacting the relevant organs, as indicated in the medical health records were considered for training. Inference was conducted on both healthy subjects and subjects with diseases. The timepoints when the participants received their imaging scans are used as CA labels. The final sample sizes of the healthy subcohorts for each organ, following manual quality control and disease-based exclusions, are summarized in Fig. 3. Following bias correction, PAGs were calculated for all individuals and organs to assess deviations from typical aging patterns. Subjects were categorized into three primary aging groups: those with accelerated aging (*PAGs* > *θ*), typical aging (∣*PAG*∣ ≤ *θ*), and decelerated aging (*PAGs* < − *θ*). The threshold *θ* was determined using the standard deviation (SD) of PAGs within the full cohort and defined separately for each organ. However, establishing a definitive threshold between typical and atypical aging remains complex due to the lack of a clear, universally accepted definition for these aging patterns.

**Fig. 4:**
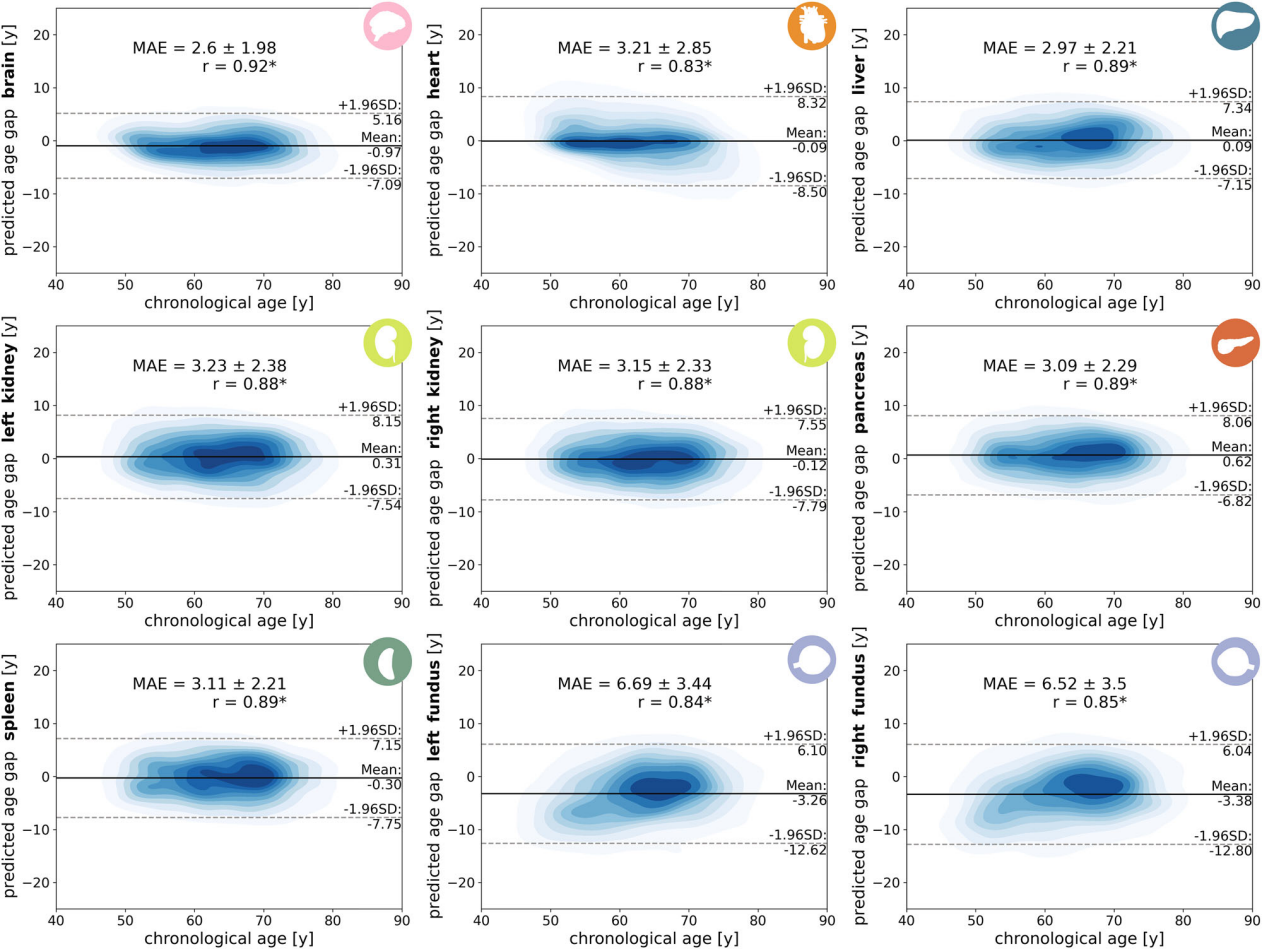
Kernel density estimations of predicted age gaps in different organs across chronological age (confidence interval = 95%) in healthy subjects (i.e., quality-controlled cohort). Mean absolute error (MAE) + standard deviation and Pearson correlation (r) between predicted and chronological ages are displayed (* indicates *p* < 5.56 × 10^−3^).

We first assessed model performance in the healthy test cohort, observing consistently strong correlations between predicted organ BA and CA across all organs. Among the studied organs, brain age demonstrated the strongest correlation and highest prediction accuracy (Pearson correlation coefficient *r* = 0.92, *p* < 1 × 10^−308^, mean absolute error (MAE) ± SD = 2.66 ± 1.98). This strong performance is likely attributed to detectable volumetric changes in white and gray matter with age^40^. In contrast, the heart and fundus showed comparatively lower performance (heart: *r* = 0.83, *p* < 1 × 10^−308^, MAE ± SD = 3.21 ± 2.85 years; left fundus: *r* = 0.84, *p* < 1 × 10^−308^, MAE ± SD = 6.69 ± 3.44 years; right fundus: *r* = 0.85, *p* < 1 × 10^−308^, MAE ± SD = 6.52 ± 3.50 years). Kernel density estimation (KDE) plots (Fig. 4) illustrate the distribution of PAGs across chronological age groups, along with confidence intervals (1.96 *SD*). These distributions confirm the feasibility of age estimation for all organs. However, for the heart, visible outliers at the youngest and oldest ends of the spectrum suggest reduced accuracy in under-represented age groups (see Fig. 2b). A similar pattern was observed for fundus age, contributing to bias and increased MAE at the age extremes.

**Fig. 5:**
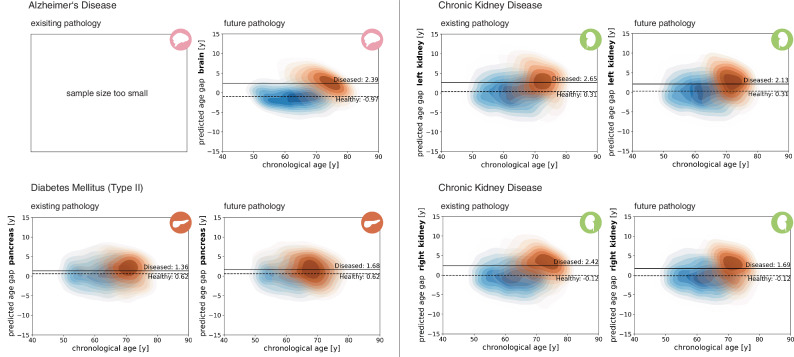
Kernel density estimations (KDE) of predicted age gaps across organs for specific health outcomes. KDE plots show predicted age gaps in test subjects with pre-existing or future diagnoses (orange) overlaid to healthy controls (blue) from Fig. 4. Mean values are shown for the healthy cohort (dashed line) and diseased cohort (solid line).

To assess the effectiveness of the selected bias correction method, we compared PAG distributions before and after correction (Fig. S4). Before correction, regression-to-the-mean bias is evident, with positive PAGs for younger participants and negative PAGs for older participants, reflecting systematic over- and underestimation of ages. This bias, however, is not equally pronounced across all organs. As noisier measurements tend to amplify regression-to-the-mean effects^41^, organs with stronger age-related signals and lower inter-individual variability, such as the brain and heart, require little correction, whereas organs with weaker signals, including the liver, spleen, pancreas, kidneys, and fundus, require greater adjustment. After correction, CA-dependent bias is effectively removed in all organs. We next tested whether predicted age gaps differed between healthy and diseased groups for a set of common diseases. Participants were stratified into three categories: (1) healthy controls, (2) individuals with an existing pathology at the time of imaging (and therefore at the time of BA estimation), and (3) individuals with a future pathology, defined as those receiving a diagnosis after the imaging appointment. KDE plots (Fig. 5) show healthy controls as a blue background distribution, with disease groups overlaid in orange. Mean values for the healthy controls and diseased subcohorts are depicted to aid interpretability. Comparisons between existing and future pathology groups were performed only when sufficient subjects were available in each group (existing Alzheimer’s disease had an insufficient sample size for this analysis). We focused on common diseases and affected organs: Alzheimer’s disease (brain), type 2 diabetes mellitus (pancreas), and chronic kidney disease (left and right kidneys). To quantitatively assess group differences, we used Welch’s t-test with Bonferroni correction (statistical significance defined as *p* < 4.55 × 10^−3^, *N* = 11 tests). In all cases of existing pathology, Welch’s t-test revealed significant differences between healthy controls and disease patients, with mean pancreas age gaps increased by 0.74 years in type 2 diabetes mellitus patients (*p* = 1.03 × 10^−10^), and mean kidney age gaps increased by 2.34 years (left, *p* = 5.18 × 10^−15^) and 2.54 years (right, *p* = 4.15 × 10^−28^) in chronic kidney disease patients. The KDE distributions showed a clear trend toward accelerated aging, with an accumulation of subjects above the healthy mean. In most cases of future pathology, this effect was also visible prior to diagnosis. Mean brain age gaps differed by 4.36 years between future Alzheimer’s disease cases and healthy controls (*p* = 6.01 × 10^−15^), mean pancreas age gaps by 0.86 years in future type 2 diabetes mellitus cases (*p* = 1.19 × 10^−10^), and mean kidney age gaps by 1.82 years (left, *p* = 9.95 × 10^−27^) and 1.81 years (right, *p* = 8.66 × 10^−23^) in future chronic kidney disease cases. Together, these results suggest that imaging-derived organ age gaps not only reflect existing pathology but may also provide early-warning biomarkers with prognostic value in clinical practice.

### Multi-organ biological age

**Fig. 6:**
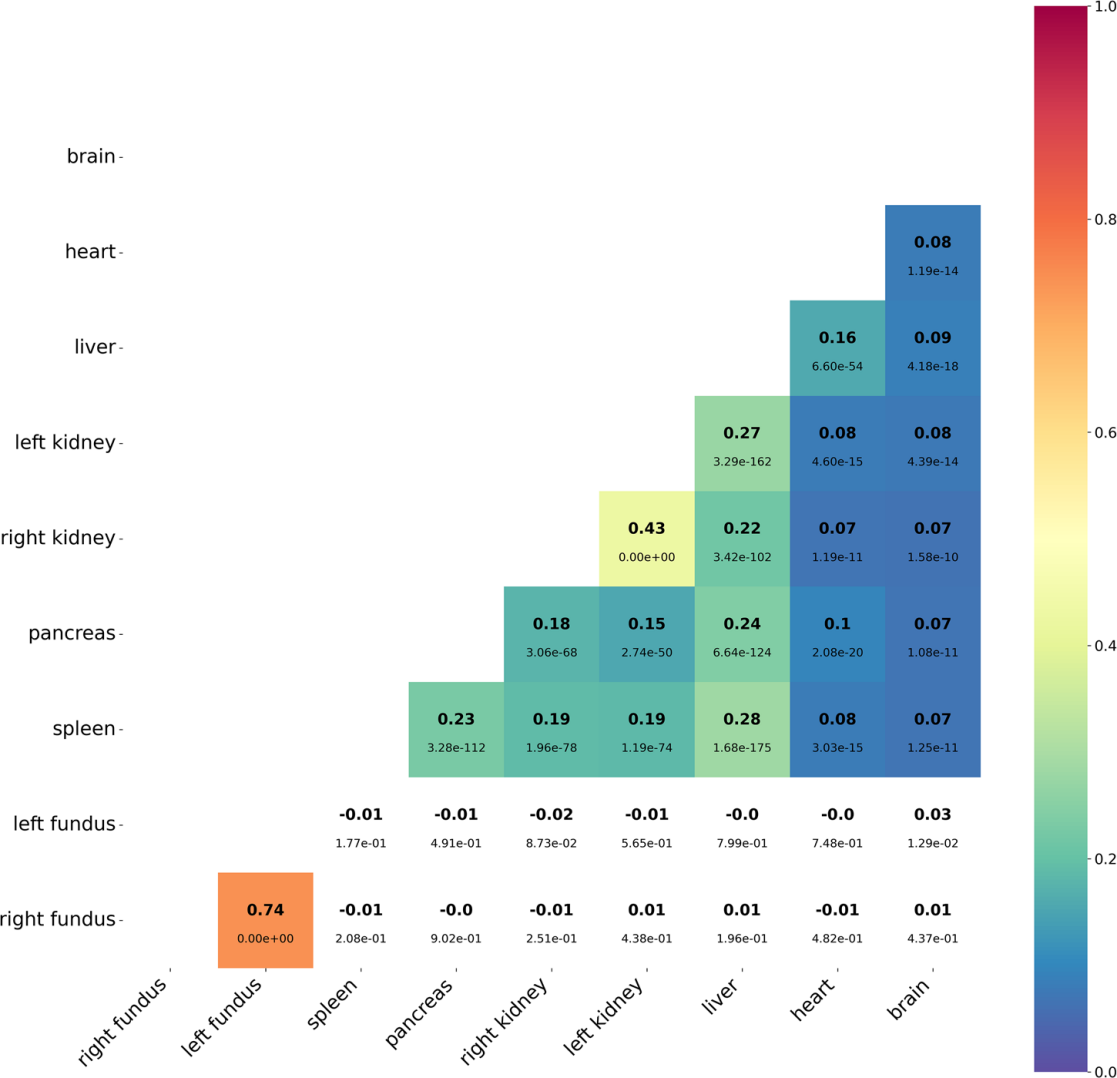
Heatmap of Pearson correlation coefficients between predicted age gaps (PAGs) of different organs. Correlation coefficients are displayed in bold above their corresponding p-values, with statistically non-significant results shown in white (Bonferroni corrected, 36 tests, *p* < 1.39 × 10^−3^).

We investigated the correlation between PAGs of different organs to assess the relationship between aging patterns across various organs. The analysis included only participants with complete imaging data across all organs (*N* = 9982, mean CA = 63.9 ± 7.73 years, 5145 female). As shown in Fig. 6, the heatmap presents Pearson correlation coefficients alongside Bonferroni-corrected *p*-values (36 tests, *p* < 1.39 × 10^−3^), with non-significant correlations omitted. Overall, PAGs were mostly independent, emphasizing the importance of organ-specific analyses. An exception was observed in bilateral organs, with considerable correlations in the kidneys (*r* = 0.43, *p* < 1 × 10^−308^) and the fundus (*r* = 0.74, *p* < 1 × 10^−308^). While recent research investigates asymmetries between bilateral organs^42^, a certain degree of correlation remains expected^43^. Therefore, these observed correlations align with biological expectations, reinforcing the validity of our PAG estimates and serving as a form of back-validation. Fundus PAGs were the most isolated, showing no significant correlations with PAGs of other organs. Similarly, brain aging appeared relatively independent, with weak correlations to other organs. In contrast, abdominal organs showed more visible associations, particularly between the liver and other abdominal organs (left kidney: *r* = 0.27, *p* = 3.29 × 10^−162^; right kidney: *r* = 0.22, *p* = 3.42 × 10^−102^; pancreas: *r* = 0.24, *p* = 6.64 × 10^−124^; spleen: *r* = 0.28, *p* = 1.68 × 10^−175^), indicating partially overlapping aging processes within these systems. Heart age gaps showed limited correlations overall, with a notable exception for liver PAGs (*r* = 0.16, *p* = 6.60 × 10^−54^).

### Explainability in BA estimation

**Fig. 7:**
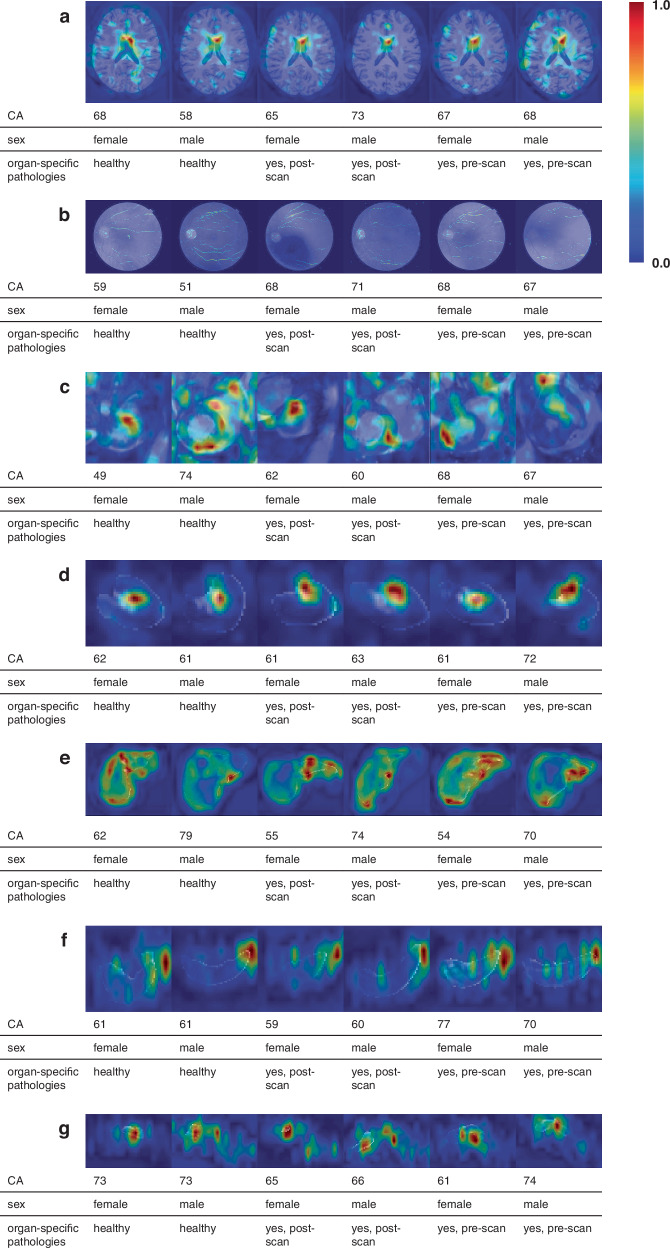
GradCAM highlights the regions influencing the model’s decisions. Representative activation patterns are shown for **a** brain, **b** fundus, **c** heart, **d** left kidney, **e** liver, **f** spleen, **g** pancreas. Color-coding indicates regions with strong (1.0) and low influence (0.0). Examples are stratified by sex and disease status.

Explainability and interpretability are crucial in estimating BA due to the lack of a true ground truth label. Grad-CAMs (Gradient-weighted Class Activation Mapping)^44^ highlight regions in the images that most influence the deep learning model, offering insights into which features drive the predictions. To validate these findings, the highlighted areas are compared to known age-related structural or functional changes, ensuring the model focuses on biologically relevant features. Figure 7 illustrates commonly observed patterns in different organs for female and male subjects, stratified by healthy status or the presence of an existing or future diagnosis (diagnosis defined here as any diagnosis that was excluded for the healthy training cohort, see Fig. S3).

In brain scans, the model often focuses on the ventricles, which are known to enlarge with age due to atrophy of surrounding brain tissue, particularly the gray matter^40,45^ (Fig. 7a). In fundus OCT scans, the model predominantly emphasizes blood vessels and the optic disc which are areas associated with retinal health and aging^46,47^ (Fig. 7b). In the heart, the model frequently highlights the myocardium and left ventricle, which undergo structural changes with aging, including ventricular dilation and thickening of the myocardial walls^48,49^ (Fig. 7c). In the kidneys, the model typically focuses on the hilum, where afferent arteriole dilation and other vascular changes are associated with aging^50^ (Fig. 7d). For the liver, aging is often accompanied by morphological changes^51,52^. While volumetric changes are difficult to represent in GradCAM analyses, the maps highlight the model’s focus on the shape and surface contours of the liver (Fig. 7e). For the spleen and pancreas, global volume loss is the primary observable age-related change. However, Grad-CAMs struggle to capture these volume changes effectively, making it difficult to identify clear and consistent patterns across different individuals (Fig. 7f, g). The highlighted features in all organs are consistent with previous reports on visual changes due to organ aging, supporting the validity of our proposed age prediction model. Overall, we observed no systematic differences in these patterns between sexes or between healthy and diseased subcohorts.

### Prognostic value of PAGs for mortality

PAGs were evaluated as potential prognostic biomarkers of diseases and mortality by comparing the likelihood of health outcomes across different aging groups using Kaplan-Meier survival analysis. Given that age is a well-established risk factor for many chronic diseases, we tested whether BA could serve as an effective biomarker for predicting disease risk and progression beyond CA. Health outcomes, including chronic diseases, acute conditions, and mortality, were selected based on their impact on organ aging and sufficient sample sizes for statistical testing. Kaplan-Meier survival curves for left-truncated and right-censored data were computed using birth as the start point, imaging appointments (starting in 2014) as the individual entry date, and the most recent data retrieval in 01/2024 as the endpoint. Additionally, we used univariate Cox proportional hazard regression to quantitatively compare survival distributions between accelerated (coded as 2), typical (coded as 1), and decelerated aging groups (coded as 0). To provide an interpretable continuous effect size, we additionally performed univariate Cox regression using continuous PAG values instead of categorical aging groups to estimate risk per 1 year increase in predicted age gap. We report the hazard ratios (HR), the confidence intervals (CI), and *p*-values. Statistical significance is defined as *p* < 2.27 × 10^−3^ (*N* = 22 tests).

**Fig. 8:**
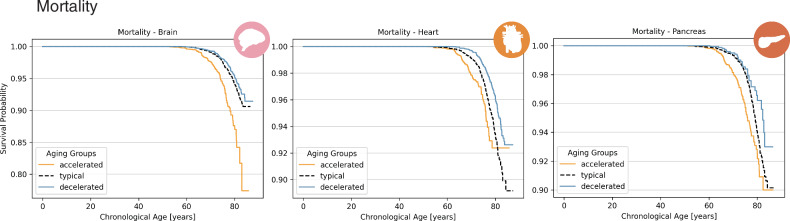
Kaplan-Meier-curves of event-free survival rate. Curves are shown for accelerated aging (*PAG* > *SD*), typical aging (∣*PAG*∣ ≤ *SD*), and decelerated aging (*PAG* < − *SD*) of the brain, heart, and pancreas, for the estimated predicted age gap (PAG).

First, we examined the impact of aging groups on survival by comparing mortality across aging categories. Figure 8 shows Kaplan-Meier survival curves for organs exhibiting significant differences in survival across aging groups, including the brain (HR: 1.77, 95% CI: 1.54–2.02, *p* = 1.41 × 10^−16^), heart (HR: 1.92, 95% CI: 1.69–2.19, *p* = 4.37 × 10^−23^), and pancreas (HR: 1.65, 95% CI: 1.38–1.96, *p* = 1.76 × 10^−8^). Individuals exhibiting accelerated aging in the brain, heart, and pancreas had a significantly higher mortality risk compared to the normative aging group, whereas those in the decelerated aging group showed a lower risk relative to typical aging. Consistent with these findings, continuous PAG analysis showed that each 1 year increase in brain PAG was associated with an estimated 13% increase in mortality risk (HR: 1.13, 95% CI: 1.10–1.16, *p* = 1.12 × 10^−18^), with similar per year risk increases observed for heart PAG (12%, HR: 1.12, 95% CI: 1.09–1.14, *p* = 7.46 × 10^−28^) and pancreas PAG (8%, HR: 1.08, 95% CI: 1.05–1.11, *p* = 4.21 × 10^−8^).

### Prognostic value of PAGs for health outcomes

**Fig. 9:**
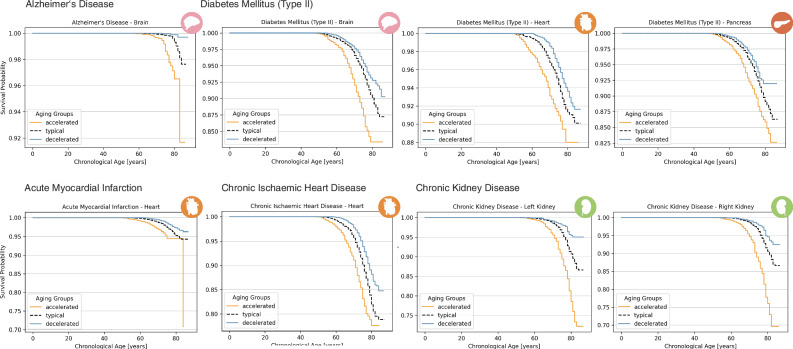
Kaplan-Meier-curves are shown for accelerated aging (*PAGs* > *SD*), typical aging (∣*PAG*∣ ≤ *SD*), and decelerated aging (*PAGs* < − *SD*) across different organs for the estimated predicted age gap (PAG) and for various diseases. Curves are shown for brain aging in patients with Alzheimer’s disease, brain, heart, and pancreas aging in type 2 diabetes mellitus, heart aging in myocardial infarction and chronic ischemic heart disease, and kidney aging in chronic kidney disease.

We also investigated the impact of PAGs on time-to-event predictions for various common health outcomes associated with specific organs (Fig. 9). For brain-related conditions, we focused on Alzheimer’s disease, which is strongly linked to accelerated brain aging. We observed a clear trend of increased risk in the advanced brain aging group, while age gaps in other organs showed no prognostic value for Alzheimer’s disease. Both the Kaplan-Meier curve and the quantitative comparison of survival probabilities across the three aging groups (HR: 5.83, 95% CI: 3.70–9.18, *p* = 2.83 × 10^−14^) reveal a significantly higher disease risk for accelerated aging and a lower risk for decelerated aging. Consistently, continuous PAG analysis showed that each 1 year increase in brain PAG was associated with an estimated 49% increase in Alzheimer’s disease risk (HR: 1.49, 95% CI: 1.36–1.62, *p* = 1.71 × 10^−19^), emphasizing brain PAGs as a strong indicator for Alzheimer’s disease. A higher incidence rate for type 2 diabetes mellitus was linked to accelerated aging across multiple organs, including the brain (HR: 1.73, 95% CI: 1.56–1.91, *p* = 4.96 × 10^−27^), heart (HR: 1.96, 95% CI: 1.78–2.16, *p* = 1.64 × 10^−42^), and pancreas (HR: 1.60, 95% CI: 1.43–1.80, *p* = 3.10 × 10^−16^). Using continuous PAGs, each 1 year increase was associated with higher diabetes risk for the brain (13%; HR: 1.13, 95% CI: 1.11–1.15, *p* = 1.02 × 10^−32^), heart (12%; HR: 1.12, 95% CI: 1.10–1.13, *p* = 3.16 × 10^−50^), and pancreas (11%; HR: 1.11, 95% CI: 1.09–1.13, *p* = 2.37 × 10^−27^), underscoring the prognostic power of organ-specific PAGs for diabetes risk.

For cardiovascular conditions, including myocardial infarction and chronic ischemic heart disease, we observed a strong, graded association between accelerated heart aging and the onset of these diseases, suggesting that accelerated heart aging is a significant risk factor for cardiovascular conditions. Comparing survival rates between accelerated, typical, and decelerated aging groups revealed a higher hazard for accelerated aging, particularly for the heart (acute myocardial infarction: HR: 2.15, 95% CI: 1.87–2.48, *p* = 2.63 × 10^−26^; chronic ischemic heart diseases: HR: 1.88, 95% CI: 1.74–2.03, *p* = 5.15 × 10^−60^). Continuous analysis showed that each 1 year increase in heart PAG was associated with a 12% increase in risk for acute myocardial infarction (HR: 1.12, 95% CI: 1.10–1.15, *p* = 4.09 × 10^−27^) and an 11% increase in risk for chronic ischemic heart disease (HR: 1.11, 95% CI: 1.10–1.12, *p* = 5.53 × 10^−75^).

Regarding kidney health, we investigated chronic kidney disease. A clear systemic trend was observed for accelerated aging in both the left and right kidney. Quantitative analysis further confirmed that individuals with accelerated renal aging had a significantly higher risk of chronic kidney disease, whereas those in the slower aging group exhibited a lower risk (left: HR: 2.60, 95% CI: 2.30–2.93, *p* = 4.16 × 10^−53^; right: HR: 2.71, 95% CI: 2.40–3.07, *p* = 7.23 × 10^−56^). Continuous PAG analysis revealed that each 1 year increase in left and right kidney PAG was associated with a 19% increase in chronic kidney disease risk, respectively (left: HR: 1.19, 95% CI: 1.16–1.21, *p* = 4.65 × 10^−63^; right: HR: 1.19, 95% CI: 1.17–1.22, *p* = 2.68 × 10^−66^).

To further evaluate whether organ-specific PAGs provide prognostic information beyond conventional risk factors, we performed a multivariable Cox analysis for type 2 diabetes mellitus, adjusting for chronological age, sex, and BMI. The likelihood ratio test indicated that including all PAGs significantly improved model fit compared with the clinical model alone (*p* = 8.26 × 10^−3^), demonstrating that imaging-derived organ aging adds independent predictive value and may complement traditional risk factors.

Together, these findings demonstrate that organ-specific predicted age gaps robustly reflect disease susceptibility, with accelerated aging consistently associated with higher risk across neurological, metabolic, cardiovascular, and renal outcomes.

## Discussion

In this study, we conducted a comprehensive imaging-based analysis of biological aging across seven organs, leveraging deep learning techniques to estimate BA from MRI and OCT imaging data. Our approach is purely image-driven, requiring no additional clinical or laboratory information. It thereby provides an objective and automated method for quantifying aging, with the potential to turn this into a fast aging screening method. A large-scale preprocessing pipeline was employed, including automatic organ segmentation, quality control, and intensity normalization. Organ-specific age regression models were trained on a healthy cohort to extract and learn aging-related features without the need for manually predefined biomarkers.

Our work represents an imaging-based quantification of aging across multiple organ systems within the whole body. This approach offers significant advantages over conventional methods using manually curated or preprocessed features, as it eliminates biases introduced by feature selection. By relying solely on imaging data, we ensure a consistent and scalable methodology applicable across large populations. The robustness of our models is further supported by their application to an extensive dataset comprising over 70,000 imaging samples from the UKB, covering the brain, heart, abdomen, and fundus. This large sample size enhances the reliability and generalizability of our BA estimates, facilitating more precise assessments of individual aging trajectories.

Our results demonstrate the feasibility of predicting BA even in organs where aging effects are not easily discernible, such as the pancreas, spleen, and liver. This highlights the sensitivity of deep learning models in capturing subtle structural and functional changes associated with aging. The brain, in particular, provided the most consistent aging patterns, likely due to its prominently observable volumetric changes in gray and white matter. Additionally, brain imaging benefits from greater homogeneity and the standardization of data through MNI152 space mapping^53^, making age regression less complex and age patterns more easily observable. To improve transparency, we utilized attention maps to visualize the imaging regions most relevant to BA predictions. These highlighted areas align with established literature on age-related organ changes, further validating the reliability of our approach.

Consistent with previous studies^18,54^, we observed significant heterogeneity in organ aging, reinforcing the necessity of multi-organ analyses. While biological age estimates for different organs were largely independent, we noted expected correlations in bilateral organs such as the kidneys and fundus. Additionally, weak but notable associations were identified among abdominal organs and between the heart and abdominal systems. These findings suggest the potential for identifying clusters of organs that share aging trajectories, which may offer new insights into systemic aging processes.

Beyond characterizing aging patterns, our study highlights the prognostic value of BA estimates. We found that accelerated aging in multiple organs serves as a strong predictor for mortality and disease outcomes. Specifically, accelerated brain aging was identified as a robust predictor for Alzheimer’s disease, while accelerated cardiac aging correlated with an increased risk of myocardial infarction and chronic ischemic heart disease. Similarly, renal aging was a significant risk factor for chronic kidney disease, and accelerated aging in various organs was associated with a higher likelihood of developing type 2 diabetes mellitus. These results underscore the clinical relevance of imaging-derived biological age estimates, potentially informing early interventions and personalized treatment strategies.

Despite the promising results, our study has several limitations. Defining thresholds for accelerated or decelerated aging remains challenging due to the absence of a universally accepted reference standard. We hypothesize that integrating clinical or laboratory biomarkers with imaging data in a multi-modal model could enhance the detection and characterization of decelerated aging while minimizing reliance on hand-crafted features. Furthermore, while our model performed well in predicting BA across a wide range of organs, additional validation in diverse, independent cohorts is necessary to confirm the generalizability of our findings. The current cohort primarily consists of participants over the age of 40 and is characterized by imbalances in ethnicity and socioeconomic status. These characteristics may bias the learned “normative aging patterns” toward those typical of overrepresented groups and introduce domain shift when applying the model to external populations. Such demographic imbalances could confound BA predictions by reflecting lifestyle, healthcare access, or disease prevalence patterns specific to these groups. Lower prediction accuracy observed for organs such as the heart and fundus at age extremes may partly reflect insufficient representation of younger or very old participants. Therefore, the current BA predictions should be interpreted with caution when applied to populations that differ substantially from the study cohort. As a next step, we plan to validate our findings in the German National Cohort study^33^, which includes younger participants (>20 years). Nevertheless additional validations are required in more diverse demographic cohorts to obtain reliable biological age biomarkers that can in the future be translated into clinical practice. Finally, the independent prognostic value of imaging-derived BA relative to established clinical risk factors remains to be systematically assessed. Conducting such comparisons will be an important next step toward establishing PAGs as robust, clinically actionable biomarkers.

While imaging-derived BA may form a biomarker, its prognostic value is not yet firmly established. Many potential confounders, including lifestyle, comorbidities, medication, and technical factors such as scanner differences, may influence predictions and need to be systematically controlled. Moreover, the extent to which imaging-derived BA provides independent prognostic information beyond conventional risk factors remains to be validated. At the same time, this represents a unique opportunity: by disentangling these confounders and systematically linking imaging-derived BA to clinical outcomes, future work can establish a more reliable and clinically actionable biomarker of aging. While much research has focused on brain aging and its links to mortality and disease, our results show that aging occurs heterogeneously across the body. Age gaps in other organs also provide meaningful prognostic information, highlighting that a multi-organ perspective can capture risk profiles more comprehensively than brain age alone. These findings underscore the value of considering organ-specific aging patterns in developing personalized prevention and treatment strategies.

In conclusion, this study presents a novel, imaging-only deep learning approach for estimating biological age across multiple organs, enabling comprehensive and objective aging assessment. Leveraging over 70,000 MRI and OCT scans from the UK Biobank, our models demonstrated strong performance in capturing aging patterns across organs, including those where age-related changes are less apparent. The resulting PAGs showed strong prognostic value for disease and mortality, highlighting their potential for clinical use and personalized health monitoring. With further validation and systematic comparison to conventional risk factors, PAGs have the potential to serve as actionable biomarkers, helping to translate imaging-derived biological age into practical tools for early detection and individualized healthcare.

## Methods

### Population study

The UKB cohort serves as a data source in this study, which entails a large-scale collection of health-related information from participants aged 44 to 83 years in the UK. In addition to collecting data on environmental and lifestyle factors, medical history, biological samples, and physical tests, an extensive imaging study has been made accessible. Written informed consent was given from all participants with ethical approval from the NHS National Research Ethics Service North West (11/NW/0382, initially granted in 2011 and renewed in 2016 and 2021), and data are handled in accordance with the Data Protection Act^55^. This work was carried out under UK Biobank Application 60520 with a data retrieval date of 01/2024. Apart from imaging data, we used non-imaging information from NHS health records, including health outcomes extracted from ICD codes, and detailed demographic data. The study complied with the principles of the Declaration of Helsinki.

### Imaging protocols

T1-weighted brain images were obtained using a 3D MPRAGE sequence on a 3T MAGNETOM Skyra scanner (Siemens Healthineers, Erlangen, Germany). For abdominal images, a 3D DIXON VIBE sequence was employed on a 1.5 T MAGNETOM Aera scanner (Siemens Healthineers, Erlangen, Germany). 2D short-axis cardiac cine MRIs were acquired as part of the UKB’s cardiovascular magnetic resonance (CMR) protocol with a bSSFP sequence on a 1.5 T MAGNETOM Aera scanner (Siemens Healthineers, Erlangen, Germany), employing electrocardiogram (ECG) gating for cardiac synchronization. Fundus images were obtained with a 3D OCT device (Topcon 3D OCT1000 Mark II, Topcon Corp., Tokyo, Japan). All MR data in the UKB are available as gray-scale magnitude images with a single input channel, except for abdominal images, which provide the four DIXON contrasts (in-phase, opposed-phase, fat, water) as input. For the fundus OCT scans, the input images consist of three RGB color input channels. An overview of important protocol parameters is given in Fig. S1.

### Data preprocessing

Automatic organ segmentation using the nnUNet^37,38^ was performed on the 3D abdominal MRIs to extract organ-focused images of the left and right kidneys, liver, spleen, and pancreas. Bounding boxes of the organs were determined to exclude irrelevant background. A tolerance margin was added to the outer boundaries of the segmentation masks. Cardiac segmentation was performed using the pre-trained model as proposed in ref. ^39^. The dimensions of the heart bounding box were determined in the end-diastolic phase. Dynamic 2D images (i.e., 2D + time) of a single slice were provided as input to the network with slice coverage over the complete heart, after image normalization to values between 0 and 1.

Aside from obtaining regional images, the segmentation framework was also used to provide initial indications of poor image quality by marking samples with strongly diverging segmentation volumes. Based on this pre-sorting, the remaining images were assessed visually with respect to their quality. No segmentation was necessary for fundus and brain images. Brain images were pre-processed by skull stripping using the FSL tool^56^ and MNI152 space mapping.

### Biological age estimation

In our previous work, we proposed an uncertainty-aware ResNet-based structure for brain age estimation^19^. To predict organ-specific ages in this work, we trained the model for all organs separately. The complete dataset was partitioned into healthy and pathological subcohorts using organ-specific ICD codes provided by the NHS, as outlined in Fig. S3. The healthy subcohort was further split into 80% for training and 20% for testing. All models were trained exclusively on the healthy training set, and performance was evaluated on both the healthy test set and the pathological subcohort. The model architecture is kept simple to avoid overconfident predictions by a too powerful architecture. As illustrated in Fig. S2, the model consists of three sequential building blocks, combining convolutional and instance normalization layers with skip connections. Three-dimensional processing blocks were selected for the MRI data, while two-dimensional layers were chosen for fundus images. Morphological and physiological variations among individuals can lead to inconsistencies in the chronological age labels. Visually similar organs may have different CAs, introducing statistical (aleatoric) and input-dependent (heteroscedastic) uncertainty into age estimates. We model this uncertainty by replacing the deterministic age predictions with a heteroscedastic Gaussian predictive distribution of the estimated age, characterized by the mean *μ*(*x*) and the logarithm of the standard deviation $\log \sigma (x)$ for a given organ image *x*. Thus, for the CA label *y* conditioned on the input image *x* and model parameters *θ* we obtain^19,57^:

1 $$\rho (y| x,\theta )={\mathcal{N}}(y;\mu (x,\theta ),\sigma (x,\theta ))$$

The standard deviation *σ*(*x*, *θ*) of the Gaussian predictive distribution signifies the degree of uncertainty, while *μ*(*x*, *θ*) represents the age estimate. The model parameters are optimized by minimizing the negative log-likelihood

2 $${\mathcal{L}}({\mathcal{B}},\theta )=\frac{1}{N}\mathop{\sum }\limits_{i=1}^{N}\frac{1}{2\sigma {({x}_{i},\theta )}^{2}}{({y}_{i}-\mu ({x}_{i},\theta ))}^{2}+\log \sigma ({x}_{i},\theta )$$

for ${\mathcal{N}}$ independent and identically distributed samples of mini-batch ${\mathcal{B}}$.

For training, 4× 48GB RTX A6000 GPUs were available. Hyperparameters were adopted from^19^ where hyperparameter optimization was performed. Sample sizes vary across organs since not all participants underwent every imaging protocol, and artifact-corrupted organ images were excluded through additional manual quality control measures. Training was performed for 200 epochs using the Adam optimizer^58^. Further training parameters are stated in Fig. S2.

### Predicted age gaps

The difference between predicted BA and CA often serves as an indicator of biological age. To accurately compute the PAGs, it is necessary to address the inherent biases in the age estimation model, known as regression to the mean bias, which tends to overestimate ages for younger individuals and underestimate ages for older individuals^59^. Traditional linear corrections model the predicted age gap solely as a function of chronological age. While effective at removing age-dependent bias, they can inadvertently remove variance linked to sex, disease status, or other biological factors, potentially masking clinically relevant differences. To address this limitation, we chose a correction method that explicitly separates the linear effect of CA from other sources of variation. To implement this, we first modeled the median of the PAGs (Δ*A*_*i*_) as a function of chronological age ${A}_{i}^{CA}$ and additional covariates (sex, existing pathology, and future pathology), whose influence we aim to preserve:

3 $${\mathrm{Median}}(\Delta {A}_{i}| {X}_{i})={\beta }_{0}+{\beta }_{1}^{\top }{X}_{i}$$

where *X*_*i*_ denotes the set of covariates. We chose median regression because the median is less sensitive to outliers than the mean, ensuring that extreme predictions do not distort the correction. Predicted ages ${\widehat{A}}_{i}^{{\mathrm{pred}}}$ were then corrected relative to a reference age ${A}_{i}^{{\mathrm{ref}}}$ (the cohort median) by subtracting the linear age effect:

4 $${\widehat{A}}_{i}^{{\mathrm{pred,corr}}}={\widehat{A}}_{i}^{{\mathrm{pred}}}-{\beta }_{{1}_{age}}\cdot [{A}_{i}^{{\mathrm{CA}}}-{A}_{i}^{{\mathrm{ref}}})]$$

where ${\beta }_{{1}_{age}}$ is the regression coefficient corresponding to chronological age. Anchoring to the reference age means that the correction is centered on the cohort median, removing systematic over- or underestimation relative to this central point. After correcting for this bias, we calculate the PAGs for all organs in each subject within the test dataset, allowing us to gain insights into the aging patterns across multiple organs for each individual.

### Survival analysis

To evaluate whether the predicted age gaps can effectively predict age-related outcomes, we employ Kaplan-Meier survival analysis. This statistical method is widely used in clinical research to assess the effectiveness of treatments by observing the survival rates over time. Typically, survival is defined by the absence of death; however, any health outcome with a distinct start date can serve as the criterion to differentiate between surviving and non-surviving cases. We exploit the Kaplan-Meier plot to analyze the differences in survival rates based on PAGs, thereby evaluating their potential as indicators for age-related health outcomes, like myocardial infarction. Instead of investigating the effects of a certain intervention, we group subjects according to their predicted age gap into accelerated (*PAG* > *SD*) and decelerated aging (*PAG* < − *SD*) and analyze the differences in survival.

First, we exclude participants who experienced the health event prior to their imaging, as our focus is on the prognostic capability of the predicted age gaps rather than retrospective detection. Consequently, our subcohort includes participants who either have not experienced the health event (event = 0) or have experienced the health outcome after their imaging scan with a clearly defined time to the event (event = 1). We use the age at imaging as the starting point for each subject and the age at our data retrieval as the study’s endpoint. Individuals who died from causes other than the targeted health event during the study period are marked as censored observations. We plot the cumulative probability of the event’s occurrence across chronological age, grouped by predicted age gaps. The rate at which the curve decreases reflects the survival duration within each group, offering insights into the influence of accelerated or decelerated aging on the likelihood of the event over time.

To quantitatively assess differences in survival between aging groups, we apply a univariate Cox proportional hazards regression model using aging group classification as the predictor variable. Aging groups are encoded as 0 (decelerated), 1 (typical), and 2 (accelerated). The analysis is performed on left-truncated and right-censored data, prepared consistently with the Kaplan-Meier analysis. In addition to categorical aging groups, we also perform univariate Cox regression using the continuous PAG as the predictor variable. This allows estimation of hazard ratios per 1 year increase in PAG, providing a more interpretable effect size for clinical risk assessment. Continuous PAG values are analyzed using the same left-truncated and right-censored setup as the Kaplan-Meier and group-based Cox analyses.

To test whether PAGs add predictive value beyond conventional clinical risk factors, we performed multivariable Cox regression adjusting for chronological age, sex, and BMI, focusing on type 2 diabetes due to its multi-organ impact. Likelihood ratio tests were used to compare models with and without PAGs, allowing assessment of their independent prognostic contribution.

## Supplementary information

Supplementary Information

## Data Availability

This research was conducted using data from the UK Biobank under application number 60520. The UK Biobank data are available to researchers upon application and approval by the UK Biobank Access Management System (https://www.ukbiobank.ac.uk/use-our-data/apply-for-access/).

## Code availability

The code used in this study is available at https://github.com/lab-midas/biological_age.
