# Supplementary material for: Imaging-derived biological age across multiple organs links to mortality and aging-related health outcomes
Source: NPJ Aging. 2026 Apr 3;12(1):51. doi: 10.1038/s41514-026-00377-7 (PMC13057220; doi:10.1038/s41514-026-00377-7)
Supplement: Supplementary file 1 — Supplementary Information [file 41514_2026_377_MOESM1_ESM.pdf]

# Supplementary Information

| | Brain | Cardiac | Abdominal | Fundus |
|---|---|---|---|---|
| **Scanner** | 3T Siemens Skyra | 1.5T Siemens Aera | 3T Siemens Skyra | Topcon OCT1000 Mark II |
| **Sequence** | 3D MPRAGE | 2D+t bSSFP CINE | 3D Dixon VIBE | - |
| **Resolution** | $1.0 \times 1.0 \times 1.0$ mm$^3$ | $1.8 \times 1.8$ mm$^2$ $\times$ 31.56 ms | $2.23 \times 2.23 \times 4$ mm$^3$ | 6 µm |
| **TE/TR** | 2.01 ms/2000 ms | 1.10 ms/2.6 ms | 2.39 ms, 4.77 ms/6.69 ms | - |
| **Flip Angle** | 8° | 80° | 10° | - |

**Fig. S1** Imaging parameters for brain, cardiac, and abdominal MRI sequences, and for OCT fundus images.

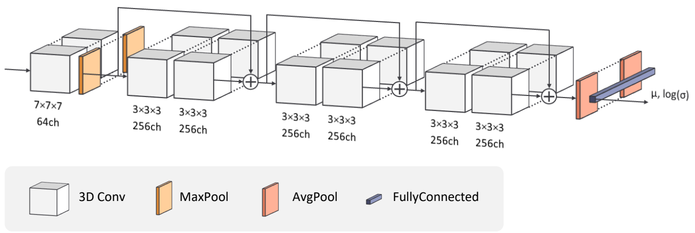


| | Brain | Fundus | Heart | Kidneys | Liver | Spleen | Pancreas |
|---|---|---|---|---|---|---|---|
| **Training Sample Size** | 29,493 | 101,144 | 37,640 | 84,504 | 43,664 | 43,922 | 43,922 |
| **Test Sample Size** | | | | | | | |
| Healthy | 6,857 | 25,287 | 9,410 | 21,126 | 9,310 | 10,981 | 10,916 |
| Pathology pre-scan | 6,998 | 12,084 | 16,059 | 16,648 | 6,998 | 6,883 | 7,137 |
| Pathology post-scan | 3,725 | 36,907 | 4,510 | 6,326 | 2,450 | 2,417 | 2,501 |
| **Optimizer** | Adam | Adam | Adam | Adam | Adam | Adam | Adam |
| **Learning Rate** | $1 \times 10^{-4}$ | $4 \times 10^{-4}$ | $1 \times 10^{-4}$ | $1 \times 10^{-4}$ | $1 \times 10^{-4}$ | $1 \times 10^{-4}$ | $1 \times 10^{-4}$ |
| **Batch Size** | 16 | 8 | 16 | 8 | 16 | 16 | 16 |
| **Number of Parameters** | 5.9 M | 1.9 M | 5.9 M | 5.9 M | 5.9 M | 5.9 M | 5.9 M |
| **Training Time** | 10d 2h 38m | 18d 16h 33m | 3d 15h 7m | 4d 10h 44m | 1mo 2d 22h 52m | 3d 2h 49m | 4d 25h 1m |

**Fig. S2** Overview of the deep learning model architecture, including relevant training parameters. The figure illustrates layer composition, channel depth, and kernel sizes for 3D processing used in MRI data. For OCT fundus images, 2D convolutional layers were applied instead.

| Brain | ICD-10 | Fundus | ICD-10 | Heart | ICD-10 | Kidneys | ICD-10 | Liver | ICD-10 | Spleen | ICD-10 | Pancreas | ICD-10 |
|---|---|---|---|---|---|---|---|---|---|---|---|---|---|
| Alzheimer Disease | G30 | Retinal vascular occlusions | H34 | Angina Pectoris | I20 | Acute renal failure | N17 | Alcoholic liver disease | K70 | Splenomegaly | R16.1 | Acute Pancreatitis | K85 |
| Dementia | F0* | Diabetic retinopathy | H36.0 | Myocardial Infarction | I21 | Chronic kidney disease | N18 | Toxic liver disease | K71 | Malignant neoplasm: Spleen | C26.1 | Malignant neoplasm of pancreas | C25 |
| Malignant neoplasm of brain | C71 | Glaucoma | H40 | Chronic Ischaemic Heart Disease | I25 | Unspecified kidney failure | N19 | Hepatic failure | K72 | Injury of spleen (Haematoma, Laceration, Rupture of spleen) | S36 | Diabeters Type 1 | E10 |
| Epilepsy | G40 | Optic neuritis | H46 | Cardiomyopathy | I42 | Acute tubulo-interstitial nephritis | N10 | Chronic hepatitis | K73 | Diseases of spleen (Hyposplenism, Hypersplenism, Abscess of spleen, Cyst of spleen, Infarction of spleen) | D73 | Diseases of Pancreas (Chronic Pancreatitis, Cyst/Pseudocyst of Pancreas) | K86 |
| Mental Disorders | F0*-F9* | Other retinal disorders (Retinopathy, Degeneration of Macula and Posterior Pole, Peripheral retinal degeneration, Hereditary retinal dystrophy, Retinal haemorrhage, Separation of retinal layers) | H35 | Cardiac Arrest | I46 | Chronic tubulo-interstitial nephritis | N11 | Fibrosis and cirrhosis of liver | K74 | Diabetes Mellitus II | E11 | Diabetes Mellitus II | E11 |
| Parkinson disease | G20 | Malignant neoplasm: Retina | C69.2 | Atrial Fibrillation | I48 | Tubulo-interstitial nephritis, not specified as acute or chronic | N12 | Other inflammatory liver diseases | K75 | Disorders of lipoprotein metabolism and other lipidaemias | E78 | Disorders of lipoprotein metabolism and other lipidaemias | E78 |
| Stroke | I63.9 | Diabetes Mellitus II | E11 | Arrhythmia | I49 | Obstructive and reflux uropathy | N13 | Hepatomegaly | R16.0 | Metabolic syndrome | E88.81 | Metabolic syndrome | E88.81 |
| Transient cerebral ischaemic attacks | G45 | Disorders of lipoprotein metabolism and other lipidaemias | E78 | Congestive Heart Failure | I50.9 | Drug- and heavy-metal-induced tubulo-interstitial and tubular conditions | N14 | Type 2 diabetes mellitus | E11 | Impaired fasting glucose | R73.1 | Impaired fasting glucose | R73.1 |
| Amytrophic Lateral Sclerosis | G12 | Metabolic syndrome | E88.81 | Carotid Artery Disease | I65 | Other renal tubulo-interstitial diseases (Balkan nephropathy, Renal and perinephric abscess) | N15 | Malignant neoplasm of liver | C22 | Hyperglycemia | R73.9 | Hyperglycemia | R73.9 |
| Ataxia | R27.0 | Impaired fasting glucose | R73.1 | Peripheral Vascular Disease | I73.9 | Cystic Kidney Disease | Q61 | Diabetes Mellitus II | E11 | | | | |
| Bell palsy | G51 | Hyperglycemia | R73.9 | Hypertension | I10 | Systemic Lupus Erythematosus | M32 | Disorders of lipoprotein metabolism and other lipidaemias | E78 | | | | |
| Meningitis | G03 | | | Hypertensive Heart Disease | I11 | Glomerular disorders | N08 | Metabolic syndrome | E88.81 | | | | |
| Guillain-Barr Syndrome | G61.0 | | | Aortic aneurysm and dissection | I71 | Haematuria | N02 | Impaired fasting glucose | R73.1 | | | | |
| Intracranial injury | S06 | | | Diabetes Mellitus II | E11 | Cystinosis | E72.04 | Hyperglycemia | R73.9 | | | | |
| Crushing Injury of Head | S07 | | | Disorders of lipoprotein metabolism and other lipidaemias | E78 | Calculus of Kidney | N20.0 | | | | | | |
| Traumatic amputation of part of head | S08 | | | Metabolic syndrome | E88.81 | Chronic tubulo-interstitial nephritis | N11 | | | | | | |
| Encephalitis | G04 | | | Impaired fasting glucose | R73.1 | Malignant neoplasm of kidney | C64 | | | | | | |
| Acute Spinal Cord Injury | S14.1 | | | Hyperglycemia | R73.9 | Diabetes Mellitus II | E11 | | | | | | |
| Cerebral Atherosclerosis | I67.2 | | | | | Disorders of lipoprotein metabolism and other lipidaemias | E78 | | | | | | |
| Ischaemic Cerebrovascular Disease | I67.9 | | | | | Metabolic syndrome | E88.81 | | | | | | |
| Diabetes Mellitus II | E11 | | | | | Impaired fasting glucose | R73.1 | | | | | | |
| Disorders of lipoprotein metabolism and other lipidaemias | E78 | | | | | Hyperglycemia | R73.9 | | | | | | |
| Metabolic syndrome | E88.81 | | | | | | | | | | | | |
| Impaired fasting glucose | R73.1 | | | | | | | | | | | | |
| Hyperglycemia | R73.9 | | | | | | | | | | | | |

**Fig. S3** Overview of excluded diagnoses per organ with corresponding ICD codes. Subjects with these conditions were removed from the training set to ensure that biological age models were trained exclusively on a healthy subcohort

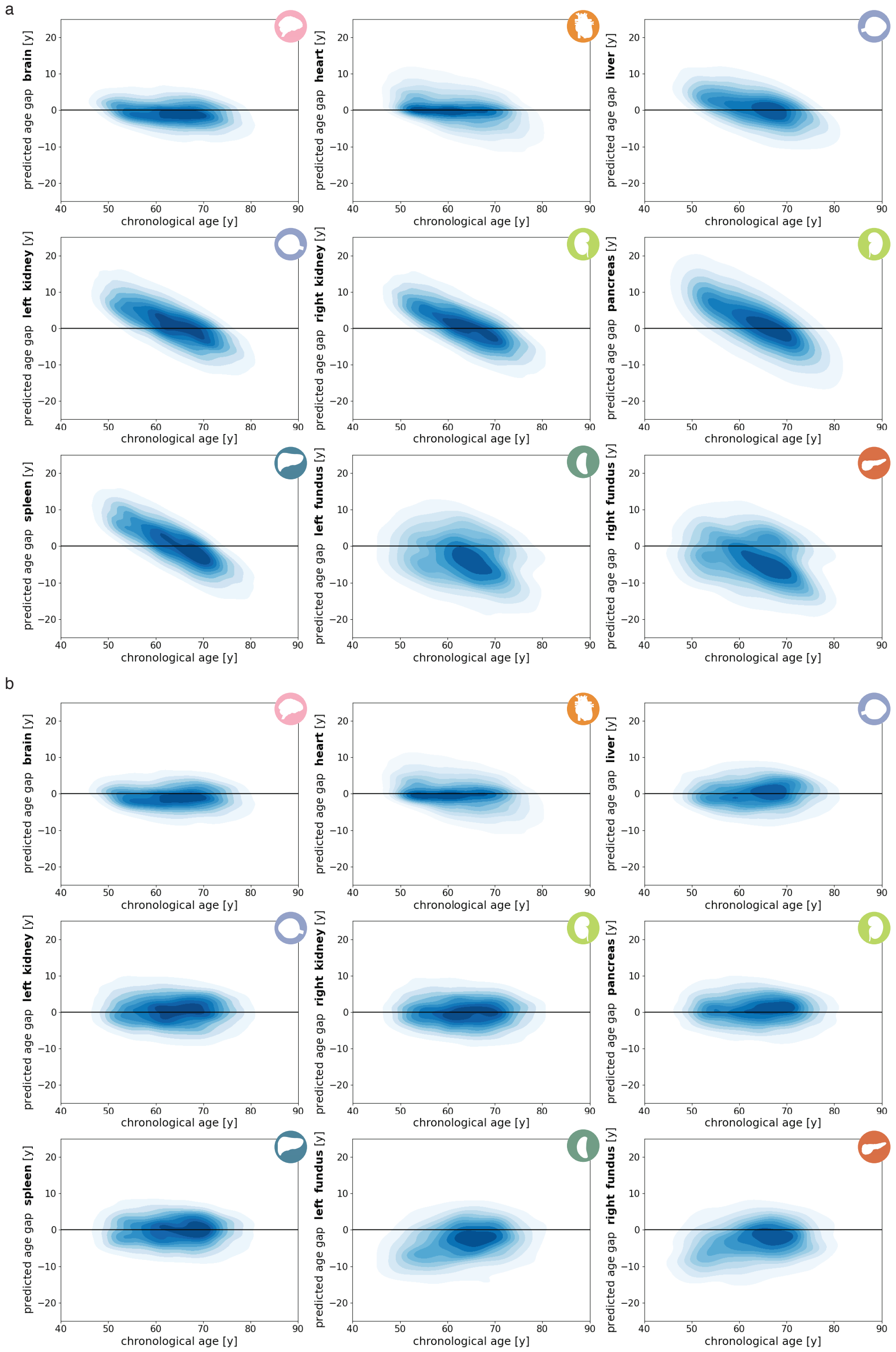


**Fig. S4** Kernel density estimations of predicted age gaps in different organs across chronological age in healthy subjects a. before and b. after bias correction. The line indicates a predicted age gap of zero.
